# Clinically significant discrepancies between sleep problems assessed by standard clinical tools and actigraphy

**DOI:** 10.1186/s12877-017-0653-7

**Published:** 2017-10-27

**Authors:** Kjersti Marie Blytt, Bjørn Bjorvatn, Bettina Husebo, Elisabeth Flo

**Affiliations:** 1 Department of Global Public Health and Primary Care, University of Bergen, Bergen, Norway; 2 Centre for Elderly and Nursing Home Medicine, University of Bergen, Bergen, Norway; 3 Norwegian Competence Center for Sleep Disorders, Haukeland University Hospital, P.O. Box 1400, 5021 Bergen, Norway; 4 Municipality of Bergen, Bergen, Norway; 5 Faculty of Psychology, University of Bergen, Bergen, Norway

**Keywords:** Actigraphy, Proxy-rating, Nursing home, Dementia, Sleep

## Abstract

**Background:**

Sleep disturbances are widespread among nursing home (NH) patients and associated with numerous negative consequences. Identifying and treating them should therefore be of high clinical priority. No prior studies have investigated the degree to which sleep disturbances as detected by actigraphy and by the sleep-related items in the *Cornell Scale for Depression in Dementia (CSDD)* and the *Neuropsychiatric Inventory – Nursing Home version (NPI-NH)* provide comparable results. Such knowledge is highly needed, since both questionnaires are used in clinical settings and studies use the NPI-NH sleep item to measure sleep disturbances. For this reason, insight into their relative (dis)advantages is valuable.

**Method:**

Cross-sectional study of 83 NH patients. Sleep was objectively measured with actigraphy for 7 days, and rated by NH staff with the sleep items in the CSDD and the NPI-NH, and results were compared. McNemar's tests were conducted to investigate whether there were significant differences between the pairs of relevant measures. Cohen's Kappa tests were used to investigate the degree of agreement between the pairs of relevant actigraphy, NPI-NH and CSDD measures. Sensitivity and specificity analyses were conducted for each of the pairs, and receiver operating characteristics (ROC) curves were designed as a plot of the true positive rate against the false positive rate for the diagnostic test.

**Results:**

Proxy-raters reported sleep disturbances in 20.5% of patients assessed with NPI-NH and 18.1% (difficulty falling asleep), 43.4% (multiple awakenings) and 3.6% (early morning awakenings) of patients had sleep disturbances assessed with CSDD. Our results showed significant differences (p<0.001) between actigraphy measures and proxy-rated sleep by the NPI-NH and CSDD. Sensitivity and specificity analyses supported these results.

**Conclusions:**

Compared to actigraphy, proxy-raters clearly underreported NH patients' sleep disturbances as assessed by sleep items in NPI-NH and CSDD. The results suggest that the usefulness of proxy-rater measures of sleep may be questionable and further research is needed into their clinical value. The results highlight the need for NH staff to acquire and act on knowledge about sleep and sleep challenges among NH patients.

**Trial registration:**

Registered at *www.clinicaltrials.gov* (registration number NCT02238652) on July 7th 2014 (6 months after study initiation).

## Background

In nursing homes (NH), wherein approximately 50–80% of patients have dementia [1–4], sleep disturbances are widespread and severe [5]. Advanced age is associated with a decrease in total sleep time [5], slow-wave sleep and rapid eye movement (REM) sleep [6]. Moreover, approximately 60% experience sleep disturbances at nighttime [7]. Disturbed sleep is associated with multiple negative consequences and predicts an increased risk of developing depression among the elderly [8]. Previous studies have shown that disturbed sleep may lead to reduced quality of life and impaired cognitive daytime functioning in elderly people with and without dementia [9, 10]. As argued by Flo et al. [11], these outcomes may be especially important for the elderly, since such symptoms may be misinterpreted as dementia or more severe dementia. Since so many institutionalized patients are affected by dementia, the consequence may be that they often are no longer able to give valid self-report, a prerequisite for adequate symptom assessment and treatment [12]. Therefore, they depend on the ability of health care professionals to evaluate and treat their distressing symptoms, including sleep disturbances.

Identifying and treating sleep disturbances in this fragile and multimorbid group should be of high clinical priority. However, evaluating sleep in NH patients with dementia is a methodological challenge [13]. Meanwhile, most tools rely primarily on interviewing NH staff members, who function as proxy-raters for the patients. This renders the reliability of such measurement uncertain [14], while their relatively low cost and effort in use, make them attractive in the clinical setting.

Wrist-worn actigraphic recordings are considered the most reliable instrument for objectively measuring sleep in this patient group [15, 16]. However, there is a high cost associated with the use of such equipment. Most et al. [17] compared the subjective assessments tools *Pittsburgh Sleep Quality Index*, *Sleep Disorders Questionnaire*, *Athens Insomnia Scale* and actigraphy. The study showed that the value of sleep questionnaires is limited in early and moderate stage Alzheimer disease and recommended actigraphy as a supplement in detecting sleep disturbances. Meanwhile, Tractenberg et al. [18] showed that scores from the *Sleep Disorders Inventory* (SDI) correlated with actigraphy data, except for 24-h total sleep time and daytime total sleep time. Hoekert et al. [19] similarly found a high degree of correlation between actigraphy and measures in the *Sleep Inventory for Normal and Pathological States*. However, the assessment tools mentioned above are not routinely used in NH settings to assess sleep. Thus, it is of high importance to investigate the accuracy of proxy-rater tools that are commonly used in both the research setting and the clinical setting, and the relative advantages and disadvantages of actigraphy and proxy-rater tools, respectively.

To our knowledge, no prior studies have investigated the relationship between clinically significant sleep disturbances as detected by actigraphy and by the sleep-related items in the *Cornell Scale for Depression in Dementia (CSDD)* and the *Neuropsychiatric Inventory – Nursing Home version (NPI-NH)*, respectively. This is highly needed, since both of the questionnaires are used in clinical settings and several studies use the NPI-NH sleep item to measure sleep disturbances among NH patients [20–23].

Consequently, the aim of this study was to investigate the degree to which actigraphy-based and proxy-rater-based assessments of sleep in NH patients provided comparable clinical outcomes. This allows for an assessment of their relative advantages and disadvantages. The study thus provides insight into similarities and differences in the measurement of sleep disturbances by means of these two approaches, which may provide crucial information for future clinical assessment procedures and research.

## Methods

### Design and setting of the study

The present study was based on baseline data from the COSMOS trial [24]; a 4-month cluster-randomized and controlled effectiveness-implementation hybrid trial with follow-up at month 9. The study was conducted in Norway from January 2014 to December 2015. To gain a representative distribution of NHs, urban/rural and big/small municipalities were invited. NH patients ≥65 years old, with and without dementia, with life expectancy >6 months, not diagnosed with schizophrenia, were eligible for inclusion. Patients with any form of chronic movement disorder or any form of paralysis in the arms/upper body were excluded from the actigraphy registrations.

### Measurements

At baseline, a research team responsible for the COSMOS trial informed and supervised NH staff in the different assessment tools. Only NH staff members who knew the patients were asked to partake in the assessment. Socio-demographic variables were collected from patients’ medical records.

Sleep was objectively assessed using the *Actiwatch Spectrum (Philips Respironics)*. Since NH patients are quite inactive, the actigraphs were placed on the patients’ dominant/mobile wrist to increase the possibility of detecting movement. Previous studies have found no difference between data collected from actigraphs placed on different locations [25, 26]. NH staff was instructed to push the event button at bed and rise times (light off in the night/light on in the morning), both by verbal and written instruction.

We used the following scoring protocols: rest intervals were set using a standardized hierarchical approach based on (1) event markers, (2) light and activity data, and (3) light or activity data. To ensure inter-scorer reliability, 30 of the actigraphy recordings were scored twice by two independent scorers, and compared in terms of total time in bed and total sleep time. To be included, participants would have to complete at least five night recordings. Sleep/wake status was determined for each one-minute epoch using the *Actiware 6* (*Respironics*) scoring program and validated algorithm, with the sensitivity set to medium. The scoring was used to generate the following variables: sleep onset latency (SOL), wake after sleep onset (WASO), early morning awakening (EMA), number of wake bouts (NoW),

To define disturbed sleep in this population we followed the quantifiable criteria described in the DSM-5 diagnostic features for insomnia [27]. Hence, we used the following cut-off points to define sleep disturbances as measured with actigraphy: SOL >30 min; WASO >30 min; EMA > 30 min. In addition, we used NoW ≥3. In accordance with Lacks and Morin [28], we used a cut-off of <85% for sleep efficiency, i.e. time spent asleep divided by time spent in bed [13].

Sleep was subjectively assessed with the NPI-NH, which is a proxy-rater inventory assessing twelve neuropsychiatric symptoms associated with dementia [29]. In the present study, we used item 11 – nighttime behavior – to ascertain sleep disturbances as observed and judged by proxy-raters. Proxy-raters were guided by questions formulated as follows: “Does the patient have sleep problems? Is s/he awake during the night? Does s/he wander during night-time, getting dressed, or going into the room of others?” Each symptom was scored for frequency (score 1–4) and severity (score 1–3), subsequently a product score was calculated thereof. In line with Garcia-Alberca et al. [20] and Chwiszczuk et al. [23], we used a product score ≥ 4 as a cut-off to define the presence of sleep disturbances.

Sleep was also assessed by the CSDD, a proxy-rater instrument for the measurement of depression, which is validated both for people with and without dementia [30–32]. Questions regarding sleep fall under the category of “cyclic functions” and comprise item 13 (“Does the patient have difficulty falling asleep?”), item 14 (“Does the patient have multiple awakenings during sleep?”) and item 15 (“Does the patient have early morning awakenings?”). For item 13, a score of 1 was given if the patient only had difficulty falling asleep a few nights in the past week and 2 if there was difficulty every night. For item 14, the patient was given a score of 1 if sleep was restless and occasionally disturbed. If the patient got out of bed in the middle of the night and/or had woken up every night in the past week, a score of 2 was given. For item 15, a score of 1 was given if the patient woke up early, but then went back to sleep. A score of 2 was given if the patient woke up earlier than usual and could not go back to sleep. A cut-off score of ≥1 was used to define sleep disturbances identified by proxy-raters for item 13 and 14. For item 15 a cut-off score of 2 was used. Item 13 was used as a measure of problems with SOL, item 14 as a measure of NoW, and item 15 as a measure of EMA, in the comparisons between the CSDD items and actigraphy measurements. The rating is in line with the guidelines by Alexopoulos et al. [30].

Cognitive function was assessed by the *Mini Mental State Examination* (MMSE), which is a 30-point validated scale that consists of 20 tasks. Scores from 0 to 10 indicate severe impairment, 11 to 20 is consistent with moderate impairment, 21 to 25 is consistent with mild impairment, and scores of 26 to 30 suggest no impairment [33, 34].

### Statistical analyses

Descriptive statistics were calculated for all relevant variables. McNemar’s tests were conducted to investigate whether or not there were significant differences between the pairs of relevant measures. Cohen’s Kappa tests were used to investigate the degree of agreement between the pairs of relevant actigraphy, NPI-NH and CSDD variables. Sensitivity and specificity analyses were also conducted for each of the pairs of measures. Furthermore, receiver operating characteristics (ROC) curves were calculated, as a plot of the true positive rate against the false positive rate for the diagnostic test. The AUC values of the ROC curves serve to evaluate the performance for each of the pairs of measures. AUC values can be assessed as follows: a value of 1 signifies a perfect test, a value of 0.97 signifies a very good test, values below 0.75 are not considered clinically useful, and values close to 0.5 have no discriminatory value at all [35].

The actigraphy measures were chosen as the reference standard and the analyses measured the degree to which the CSDD and NPI-NH measures captured the same as did the actigraphy measures. To test whether the final actigraphy sample (*n* = 83) differed systematically from the remainder of the study sample (*n* = 462), we conducted independent samples t-tests comparing the mean scores of the two samples for the following variables: age, sex, MMSE score, CSDD scores (difficulty falling asleep; early morning awakening; multiple awakenings) and NPI-NH score (sleep item). We conducted the statistical analyses using *IBM SPSS Statistics 22*.

### Ethics

Informed written consent was obtained through direct conversation with patients. If the patient lacked the ability to give consent, we obtained it through direct conversation with the patient’s legal guardian. The legal guardian gave presumed consent on behalf of the patient. This is in line with local legislation. The trial was approved by the Regional Committee for Medical and Health Research Ethics, West Norway (REK 2013/1765) and registered at www.clinicaltrials.gov (NCT02238652).

## Results

A total of 700 NH patients were invited to participate in the COSMOS study, of which 545 participants from 67 NH units were included. The first 10 patients in every NH unit were evaluated for inclusion in the actigraph subproject. The actigraphy subproject included 107 patients, 24 of whom were excluded due to actigraph malfunction or because of missing data. The final sample thus included 83 patients who wore actigraphs and had complete CSDD and NPI-NH scores. For the variables outlined above, there were no statistically significant differences between the scores for the actigraphy sample and the remainder of the study sample. Patient characteristics are summarized in Table 1.

**Table 1:** The table shows descriptive statistics on prevalence (mean values and standard deviations) for socio-demographic variables, NPI-NH^1^ sum score = Neuropsychiatric Inventory – Nursing Home version, CSDD^2^ = Cornell Scale for Depression in Dementia. MMSE^3^ = Mini Mental State Examination. SD = standard deviation

Descriptive statistics (*n* = 83)	
Age (mean, SD)	86.6 (8.1)
Female (percentage)	75.9% (*n* = 63)
CSDD^2^ sum score (mean, SD)	6.0 (5.9)
MMSE^3^ sum score (mean, SD)	10.4 (7.5)
MMSE score 20 or below (number, percentage)	59 (86.8%)
MMSE score > 20 (number, percentage)	9 (13.2%)
Number of medications (mean, SD)	7.5 (3.7)

### Sleep disturbances in NH patients as assessed by actigraphy

The mean number of actigraphy-registered nights per patient was 6.6 (SD = 1.1). Mean time spent in bed was 12 h and 20 min (SD = 1 h 43 min). Mean sleep efficiency was 64.1% (SD = 19.2), and 89.2% of the patients had sleep efficiency <85%. Mean SOL was 57.9 min (SD = 80.1) and 45.8% had SOL >30 min. Mean WASO was 151.8 min (SD = 80.2), i.e. approximately 2.5 h, and 97.6% had WASO >30 min. Mean EMA was 54.5 min (SD = 66.5), and 59.0% of the patients had EMA > 30 min. Mean NoW was 32.1 (SD = 13.4), with a mean length of 5.1 min (SD = 3.1). All actigraphy results are summarized in Table 2.

**Table 2:** Actigraphically measured sleep parameters, mean values with standard deviations

Sleep parameters (*n* = 83)	
Time spent in bed (hours:min), mean (SD)	12:20 (1:43)
Observation nights, mean (SD)	6.6 (1.0)
Sleep efficiency (%), mean (SD)	64.1 (19.2)
Sleep efficiency below 85%	89.2% (*n* = 74)
SOL (min), mean (SD)	57.9 (80.1)
Patients with SOL above 30 min	45.8% (*n* = 38)
WASO (min), mean (SD)	151.8 (80.2)
WASO above 30 min	97.6% (*n* = 81)
EMA (min), mean (SD)	54.5 (66.5)
EMA above 30 min	59.0% (*n* = 49)
NoW mean (SD)	32.1 (13.4)
NoW equal or above 3	98.8% (*n* = 82)
Length of wake bouts (min), mean (SD)	5.1 (3.1)
Bedtime (hours:min), mean (SD)	20:20 (2.21)
Wake up time (hours:min), mean (SD)	8:57 (1:29)

SOL refers to sleep onset latency. WASO refers to wake after sleep onset. EMA refers to early morning awakening. NoW refers to the number of wake bouts

### Sleep disturbances assessed with NPI-NH compared with actigraphy

Proxy-raters reported sleep disturbances in 20.5% of patients assessed with NPI-NH. McNemar’s test comparing sleep efficiency measured with actigraphy and proxy-rater sleep (NPI-NH-SS ≥ 4) showed a significant difference (*p* < 0.001) between the measures (see Table 3). This was supported by the Cohen’s Kappa analysis, which showed very low agreement between the measures (k = .029).

**Table 3:** Significant differences between actigraphy measured wrist activity compared to percentages of patients’ sleep outcome measured with proxy-rated CSDD and NPI-NH

	Actigraphy	CSDD	NPI-NH	*p*	k
SOL above 30 min or CSDD-DFA ≥ 1	45.8% (*n* = 38)	18.1% (*n* = 15)		<0.001	.105
EMA above 30 min or CSDD-EMA ≥ 2	59.0% (*n* = 49)	3.6% (n = 3)		<0.001	.051
NoW ≥3 or CSDD-MA ≥ 1	98.8% (*n* = 82)	43.4% (*n* = 36)		<0.001	.019
SE below 85% or NPI-NH-SS ≥ 4	89.2% (*n* = 74)		20.5% (*n* = 17)	<0.001	.029

SOL refers to the Sleep Onset Latency measure using actigraphy. EMA refers to the Early Morning Awakening measure. NoW refers to the Number of Wake Bouts measure. SE refers to the Sleep Efficiency measure. CSDD-DFA refers to the Difficulty Falling Asleep measure in the Cornell Scale for Depression in Dementia (CSDD). CSDD-EMA refers to the Early Morning Awakening measure in the CSDD. CSDD-MA refers to the Multiple Awakenings measure in the CSDD. NPI-NH-SS refers to the Subjective Sleep measure in the Neuropsychiatric Inventory – Nursing Home version. k = Cohen’s Kappa

In the NPI-NH measurements, we found one false positive (i.e. instances where proxy-raters reported sleep disturbances when actigraphy did not) and 57 false negatives (i.e. instances where proxy-raters did not report sleep disturbances when actigraphy did). Compared with the sleep efficiency measure, the sensitivity of the NPI-NH proxy-rater sleep measure was 21.9% (95% CI = 13.4% - 33.4%). The specificity of the measure was 88.9% (95% CI = 50.7% - 99.4%). Thus, the positive likelihood ratio of the test was 1.97, while the negative likelihood ratio of the test was 0.88. The AUC value of the ROC curve was 0.554.

### Sleep disturbances assessed with CSDD compared with actigraphy

McNemar’s test for actigraphy SOL >30 min (45.8%) and the CSDD “difficulty falling asleep” (18.1%) item showed a significant difference (*p* < 0.001) between the measures (see Table 3). This was supported by the Cohen’s Kappa analysis, which showed very low agreement between the measures (k = .105). In the CSDD SOL measurements, there were six false positives and 29 false negatives. Compared with the actigraphy measure, the sensitivity of the CSDD “difficulty falling asleep” measure was 23.7% (95% CI = 12.0% - 40.6%). The specificity of the CSDD was 86.4% (95% CI = 72.0% - 94.3%). Thus, the positive likelihood ratio of the test was 1.74, while the negative likelihood ratio of the test was 0.88. The AUC value of the ROC curve was 0.550.

McNemar’s test comparing EMA > 30 min measured with actigraphy (59%) and the CSDD “does the patient have early morning awakenings?” (EMA) item (3.6%) showed a significant difference (*p* < 0.001) between the measures (see Table 3). This was supported by the Cohen’s Kappa analysis, which showed very low agreement between the measures (k = .051). In the CSDD EMA measurements, there were no false positives, but 46 false negatives. Compared with the actigraphy measure, the sensitivity of the CSDD EMA measure was 6.1% (95% CI = 1.59% - 17.9%). The specificity of the measure was 100% (95% CI = 87.4% - 100%). Thus, the positive likelihood ratio of the test cannot be calculated, while the negative likelihood ratio of the test was 0.94. The AUC value of the ROC curve was 0.531.

McNemar’s test comparing NoW ≥3 measured with actigraphy (98.8%) and CSDD “multiple awakenings during sleep” item (43.4%) showed a significant difference (*p* < 0.001) between the measures (see Table 3). This was supported by the Cohen’s Kappa analysis, which showed a very low agreement between the measures (k = .019). In the CSDD NoW measurements, there were no false positives, but 45 false negatives. Compared with the NoW as measured by actigraphy, the sensitivity of the CSDD “multiple awakenings during sleep” measure was 44.4% (95% CI = 33.5% - 55.9%). The specificity of the measure was not possible to calculate, due to the low number of observations. Thus, the positive likelihood ratio cannot be calculated, but the negative likelihood ratio of the test was 0.56. The AUC value of the ROC curve was 0.722.

## Discussion

The aim of this study was to investigate the degree to which actigraphy-based and common proxy-rater-based assessments of sleep in NH patients provided comparable clinical outcomes. This allows for an assessment of their relative merits, when the costs, efforts and benefits of their use are taken into account. Taken together, the analyses (McNemar’s test, Cohen’s Kappa and sensitivity/specificity analyses, all of which are reported in Table 3) revealed that there were highly significant differences (*p* < 0.001) between the measures with respect to their ability to capture the various sleep outcomes (SOL, EMA and NoW). The Cohen’s Kappa values suggested low degrees of agreement between the measures for all pairs of variables. This was also supported by the sensitivity, specificity and likelihood ratio analyses, and the corresponding ROC-curves. The results overall revealed that the CSDD and NPI-NH measures had from very small to small probability for capturing the sleep outcomes detected by actigraphic recordings. This is of key importance since it implies that sleep disturbances may go undetected and thereby untreated among NH patients. These results should be viewed in the context of the nature of the two measures: While actigraphy involves the use of equipment which implies relatively high cost in use, proxy-rater tools are used mostly for screening purposes with low cost and effort.

Using NPI-NH, staff categorized 20.5% of the patients as having sleep disturbances. This was significantly lower than the objective actigraphy measure of sleep, by which 89.2% had sleep efficiency below 85%. Since the study included both patients with and without dementia, it is important to notice that the NPI-NH was developed for use among people with dementia. However, in the total sample, 87% of patients had an MMSE score < 20, which is compatible with dementia [34]. Only 13% had an MMSE score > 20, and the mean MMSE score in this sub-group was 23.6. Based on this, we can assume that most of the patients in the total sample have mild cognitive impairment or dementia. For this reason, we have included the NPI-NH scores of all patients in the present study. Comparing sleep efficiency with the NPI-NH sleep item is not optimal, since sleep efficiency is a measure of time spent asleep divided by time spent in bed, while the NPI-NH more broadly captures general sleep disturbances. However, sleep efficiency is often used as an indicator of sleep quality [36, 37]. Thus, it can be argued that the sleep item in NPI-NH to some extent should capture sleep quality and/or disturbances. The excessive time in bed reported in our study, which is an important determinant for the calculation of sleep efficiency, is in accordance with previous studies [13, 16].

Actigraphy detected significantly more sleep disturbances relating to SOL, NoW and EMA than did CSDD sleep items. These results thus also indicate that NH staff underreport or do not recognize patients’ sleep difficulties, as captured by actigraphy. In contrast, Fetveit and Bjorvatn [13] found that NH staff observations (diaries) of SOL and EMA were consistent with actigraphic recordings. However, the way these parameters are measured is not comparable with the measurements of the present study. NH staff diaries are based on observation during a given period, and the observation is recorded in writing. It is noteworthy, however, that nocturnal awakenings registered by NH staff in the study by Fetveit and Bjorvatn [13] showed little correlation with actigraphy-recorded WASO. This is in line with the present findings, which also indicated that NH staff noticed fewer awakenings compared with actigraphy.

Is the divergence between the actigraphy recordings and proxy-rater assessments due to the raters or due to the rating instruments? A potential reason could be lack of knowledge about sleep among NH staff. This could in turn result in lower perceptiveness in recognizing sleep disturbances. In addition, the proxy-raters were not necessarily night workers. It is possible that observations from night workers were not properly conveyed to the day shift staff. Furthermore, many patients in Norwegian NHs lie in bed during night-time with the cot side of the bed in the upward position. The consequence is that many patients are unable to exit the bed at night. Combined with a reduced capacity for verbal expression due to dementia, this may reduce their interaction with the night shift workers, which could lead to an impression of sleeping even when patients might be awake.

In line with previous research, the results of the present study showed that sleep disturbances are very common among NH patients. Interestingly, the findings indicate that sleep disturbances as measured with actigraphy are even more prevalent now than what was found in earlier studies. Fetveit and Bjorvatn [13] found mean sleep efficiency of 75% among NH patients, with 72% of the patients displaying sleep efficiency below 85%. A pioneering study by Ancoli-Israel et al. [38] found that patients on average slept 39.5 min per hour in any hour of the night, and 50% woke up 2 to 3 times per hour. The patients in the present study displayed a mean sleep efficiency of 64% and as many as 89.2% of the patients had sleep efficiency below 85%.

It is beyond the scope of this study to explore the discrepancy between results regarding actigraphy sleep parameters herein and results from earlier studies. However, a recent report shows that the proportion of NH patients with comprehensive assistance needs has increased from 2009 to 2015. This suggests that the NH population is generally in poorer condition now than earlier [39]. This is notable since previous studies have shown that a decreased ability to sleep is associated with comorbidities [40]. This development may potentially explain some of the discrepancy between prior studies and the present study.

The sample size of 83 patients with actigraphy assessment in the present study is larger than previous studies using actigraphy to assess sleep in this population [13, 16, 41, 42]. The low agreement between actigraphy and proxy-rater measures may simply indicate that the CSDD and the NPI-NH fail to capture sleep difficulties. In light of recent research that indicates that when the CSDD is administered by NH staff, its clinical utility is highly questionable, the discrepancy found in the present study also questions the use of proxy-raters to ascertain symptoms [43]. However, it is noteworthy that we do not recommend actigraphy as the primary tool for evaluating sleep in the NH setting. This would arguably be costly and time consuming, and thus not feasible as a screening tool. However, the results are suggestive of a need for more precise instruments for measuring sleep among NH patients, which could be used in a low-cost and valid manner by proxy-raters.

### Limitations

Previous studies indicate that actigraphy is less accurate in distinguishing sleep from wakefulness when sleep efficiency is reduced [22, 35]. Therefore, actigraphy recordings may overestimate sleep relative to sleep diaries and polysomnography [44, 45]. Taking this into consideration, the total amount of sleep may be less and even more fragmented than what is suggested by the results from the present study. This means that the sensitivity for sleep in the NPI-NH and CSDD may be even lower than estimated herein. Meanwhile, polysomnography is not an optimal form for assessing sleep in this patient population. It is difficult to score since electroencephalography does not produce clear patterns of sleep stages in demented patients [15]. Secondly, there is a low tolerance in this group for wearing such equipment [13]. Actigraphy is therefore considered the best method for assessing sleep objectively in this population [15, 16].

## Conclusion

The study revealed that when sleep was measured with common clinical tools like NPI-NH and CSDD, sleep disturbances were clearly underreported or unrecognized by NH staff as compared with actigraphy. The results thus suggest that the usefulness of proxy-rater measures of sleep may be questionable and further research is needed into its clinical value. Our results do not allow us to conclude whether the divergence in results are due to the raters or the rating instruments. However, in order to enable NH staff to treat sleep disturbances, the first step is to identify that the patient has a problem. The results therefore highlight the need for NH staff to acquire and act on knowledge about sleep and potential sleep challenges in the population of NH patients, which in turn may increase the likelihood for adequate treatment.

## Abbreviations

CSDD: Cornell Scale for Depression in Dementia
EMA: Early morning awakening
MMSE: Mini Mental State Examination
NH: Nursing Home
NoW: Number of wake bouts
NPI-NH: Neuropsychiatric Inventory – Nursing Home version
NPS: Neuropsychiatric symptoms
REM: Rapid eye movement
ROC: Receiver operating characteristics
SDI: Sleep Disorders Inventory
SOL: Sleep onset latency
WASO: Wake after sleep onset
